# Spatial patterns of pathogen prevalence in questing *Ixodes ricinus* nymphs in southern Scandinavia, 2016

**DOI:** 10.1038/s41598-020-76334-5

**Published:** 2020-11-09

**Authors:** Lene Jung Kjær, Kirstine Klitgaard, Arnulf Soleng, Kristin Skarsfjord Edgar, Heidi Elisabeth H. Lindstedt, Katrine M. Paulsen, Åshild Kristine Andreassen, Lars Korslund, Vivian Kjelland, Audun Slettan, Snorre Stuen, Petter Kjellander, Madeleine Christensson, Malin Teräväinen, Andreas Baum, Laura Mark Jensen, René Bødker

**Affiliations:** 1 Department of Veterinary and Animal Sciences, Faculty of Health and Medical Sciences, University of Copenhagen, Frederiksberg, Denmark; 2 Department for Diagnostics and Scientific Advice, National Veterinary Institute, Technical University of Denmark, Lyngby, Denmark; 3 Department of Pest Control, Norwegian Institute of Public Health, Oslo, Norway; 4 Department of Virology, Norwegian Institute of Public Health, Oslo, Norway; 5 Department of Production Animal Clinical Sciences, Norwegian University of Life Sciences, Oslo, Norway; 6 Department of Natural Sciences, University of Agder, Kristiansand, Norway; 7 Research Unit, Sørlandet Hospital Health Enterprise, Kristiansand, Norway; 8 Department of Production Animal Clinical Sciences, Section of Small Ruminant Research, Norwegian University of Life Sciences, Sandnes, Norway; 9 Department of Ecology, Grimsö Wildlife Research Station, Swedish University of Agricultural Sciences, Riddarhyttan, Sweden; 10 Department of Applied Mathematics and Computer Science, Technical University of Denmark, Lyngby, Denmark

**Keywords:** Computational biology and bioinformatics, Machine learning, High-throughput screening, Microbiology techniques, Ecological epidemiology

## Abstract

Tick-borne pathogens cause diseases in animals and humans, and tick-borne disease incidence is increasing in many parts of the world. There is a need to assess the distribution of tick-borne pathogens and identify potential risk areas. We collected 29,440 tick nymphs from 50 sites in Scandinavia from August to September, 2016. We tested ticks in a real-time PCR chip, screening for 19 vector-associated pathogens. We analysed spatial patterns, mapped the prevalence of each pathogen and used machine learning algorithms and environmental variables to develop predictive prevalence models. All 50 sites had a pool prevalence of at least 33% for one or more pathogens, the most prevalent being *Borrelia afzelii, B. garinii*, *Rickettsia helvetica*, *Anaplasma phagocytophilum,* and *Neoehrlichia mikurensis*. There were large differences in pathogen prevalence between sites, but we identified only limited geographical clustering. The prevalence models performed poorly, with only models for *R. helvetica* and *N. mikurensis* having moderate predictive power (normalized RMSE from 0.74–0.75, R^2^ from 0.43–0.48). The poor performance of the majority of our prevalence models suggest that the used environmental and climatic variables alone do not explain pathogen prevalence patterns in Scandinavia, although previously the same variables successfully predicted spatial patterns of ticks in the same area.

## Introduction

Ticks are blood-sucking arthropods and transmit a wide range of disease-causing pathogens impacting both humans and animals^1–3^. They are capable of transmitting bacteria, viruses and protozoa, to their vertebrate hosts via blood meals, and the diseases caused by these etiological agents affect both animals and humans^1,4^. Tick-borne diseases have increased in incidence and geographical range over the last decades^4–6^ and previously, 30 European Ministries of Health have recognised vector-borne diseases as the “biggest threat arising from environmental change”, with tick-borne encephalitis (TBE) and Lyme borreliosis (LB) ranking high on the list^7,8^. Predicted temperature increase, resulting from global warming^9^, could potentially cause tick range expansions, prolonged season of tick activity, changes to tick development rate and reproduction as well as the development rates of the pathogens these vectors may carry^10–12^. In Scandinavia, LB and TBE show an increasing trend, particularly in Norway and Sweden^13–16^, giving rise to public health concerns.

As ticks go through the different life stages, they feed on a variety of species such as birds, mammals and reptiles^12^. In Scandinavia, the dominating vector for disease-causing pathogens in humans is the castor bean tick, *Ixodes ricinus*^6,17^. A generalist, this hard tick is a parasite of over 300 different host species, exposing it to reservoirs of multiple pathogens^18–20^. Immature life stages of *I. ricinus* feed on hosts of all sizes, whereas adult stages mainly feed on larger hosts, making ungulates and livestock important for maintaining these tick populations, also as a means for geographical dispersal^12^. Long distance dispersal of ticks and their associated pathogens by migrating passerine birds have been documented in several studies from Scandinavia^21,22^.

LB causing pathogens belong to the *Borrelia burgdorferi* sensu lato (s.l.) species complex, with the most common in southern Scandinavia being *B. afzelii*, *B. garinii*, *B. burgdorferi* sensu stricto (s.s.), *B. spielmanii* and *B. valaisiana,* with *B. lusitaniae* being relatively rare^4,23–27^. Species belonging to the *B. burgdorferi* s.l. complex are some of the main disease causing tick-borne pathogens in the northern hemisphere, and clinical manifestations differ between the different pathogen species^28–30^. Other disease-causing pathogens reported from the region are *B. miyamotoi*, *Anaplasma phagocytophilum*, *Rickettsia helvetica*, *Neoehrlichia mikurensis*, *Babesia divergens*, *B. microti*, *B. venatorum*, *Bartonella henselae*, and TBE-virus complex (TBEV)^4,31–37^. *A. phagocythopilum* and the different *Babesia* species have long been known to cause disease in domestic livestock^33^, but may also cause disease in humans^38–40^. Although rarely, *B. miyamotoi*, *R. helvetica* and *N. mikurensis* have also been reported to cause disease in humans in southern Scandinavia^41–44^.

The risk of transmission of pathogens not only depends on the tick vector but also on reservoir hosts as some pathogens are associated with particular vertebrate species, whereas others are more generalists^35^. Thus, host species community composition may determine which pathogens are present and how prevalent they are. Within the *B. burgdorferi* s.l. species complex found in Scandinavia, most of the species appear to have rodents as their main reservoir hosts^45^. However, *B. garinii* and *B. valaisiana* have been found to be associated with birds^19,45^, and *B. lusitaniae* is more prevalent in certain lizard species (*Lacerta agilis, Podarcis muralis*)^19,45^. *B. miyamotoi* has mostly been associated with rodents^18^, whereas reservoir hosts for TBEV are still debated, but thought to be mainly rodents, small mammals and the tick vector themselves^18,45,46^. Rodents and wild ruminants have been found to be major reservoirs of *A. phagocytophilum* in the US and Asia ^33,47,48^*.* Although found in rodents in Europe, wild ruminants seems to be the main reservoir^33^. Reservoir hosts for *N. mikurensis* have not yet been fully investigated, but is thought to be wild rodents^49–51^. The different *Babesia* species have different reservoirs, with *B. divergens* associating with bovines, *B. microti* with rodents and *B. venatorum* with cervids^52^. Although rodents and other mammals seem to be a reservoir for *R. helvetica*, the ticks themselves also serve as a major reservoir, with transovarial and sexual transmission^18,53^.

Prevalence of a given pathogen may not only be determined by the availability of suitable host species. Several studies have found a correlation between pathogen prevalence and tick abundance^54–57^, which in turn is affected by several factors, such as climate^1,5^, land cover, landscape composition, and availability and density of host species^5,12^. Climate and landscape may directly affect ticks and their life cycle^1,5^, but they may additionally affect ticks indirectly as these factors also influence their host species. *I. ricinus*, for example, have been found to have high abundance in forested habitats that provide optimal conditions for tick survival^58–60^. These forested habitats also provide optimal conditions for tick host species, particularly forest edge zones where the availability of both food and shelter can elevate local abundance of several host species^60,61^. Thus, the prevalence of specific pathogens in a region is a result of the complex interaction between ticks, their host species and the environment.

For vector-borne diseases it may be important to define zones with high pathogen prevalence as they pose a health-risk to both people and animals living within these areas^62^. Maps of disease prevalence can help pin-point high risk areas and finding geographical patterns within these maps may aid in determining causality or predicting potential outbreak areas^63^. The complex nature of tick-borne pathogen transmission cycles can complicate the development of predictive models of pathogen prevalence, as it can be difficult to obtain and include important factors driving these cycles. However, as environmental variables may affect both ticks, their hosts and habitat, environmental data can potentially be used directly and as proxies in predictive models^64^. Numerous studies have linked biotic and abiotic data to the prevalence of tick-borne pathogens^64–66^. Stefanoff et al.^66^ used temperature, precipitation, and various variables related to forest cover and unemployment rates in humans to predict high risk TBE areas in Poland, and Hönig et al.^64^ used environmental data to predict probability of tick infection for both LB and TBE in the Czech Republic and Germany. Randolph and Rodgers^65^ used pattern matching modelling to relate climatic variables to the distribution of TBEV in mainland Europe, Scandinavia and the Baltic States and then projected this model to future climate change scenarios.

In previous studies, we have successfully modelled the distribution and abundance of *I. ricinus* ticks in Scandinavia using predictive machine learning (ML) algorithms and environmental variables^67,68^. Applying the same methods to data on tick-borne pathogens, may enable us to create predictive models for pathogen prevalence for southern Scandinavia, contributing to our understanding of the variation we find in human disease incidence. The resulting risk maps from such models could help identify areas at risk for tick-borne disease transmission. Here we present data on prevalence for 15 different tick-borne pathogens in southern Scandinavia. The data stems from the first coordinated multi-country study in northern Europe to date, with 29,440 tick nymphs collected from 50 sites in Denmark, Norway and Sweden and are available from the figshare repository^69^. We also present predictive maps of pathogen prevalence within southern Scandinavia, developed using ML algorithms and environmental variables.

## Methods

### Study region, site selection and field study

**Figure 1:**
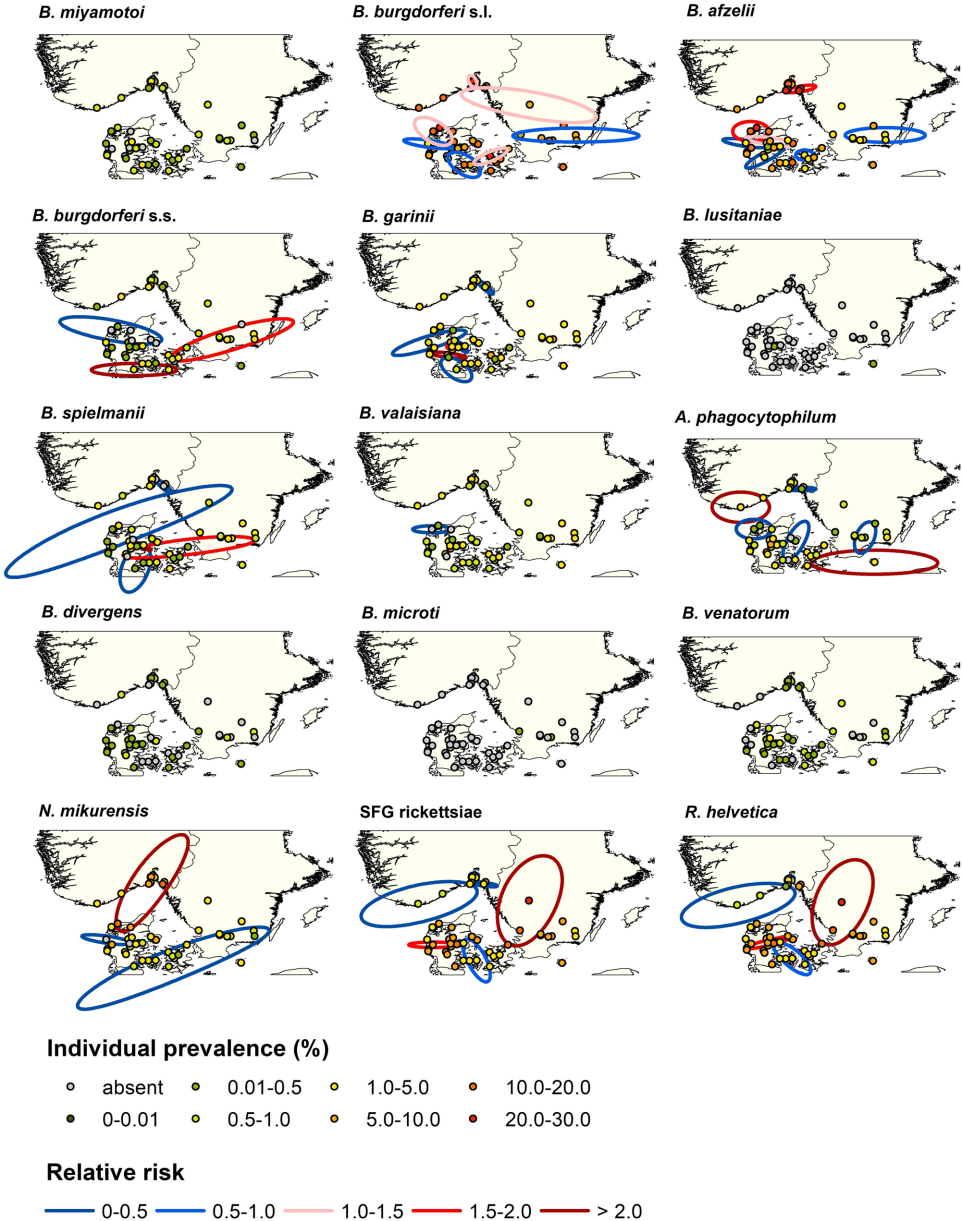
Individual prevalence. Individual prevalence at the 50 sample sites for the 15 pathogens found within the study region. Individual prevalence was calculated using method 3 from Cowling et al.^77^ that assumes 100% test sensitivity and specificity and fixed pool size. Clusters were analysed using SatScan on pool prevalence, and only significant clusters with the maximum Gini coefficient are depicted. Pool prevalence was calculated as positive pools out of the total number of pools at each site, whereas relative risk is calculated by SatScan as the estimated risk within a cluster divided by the estimated risk outside the cluster.

The stratification of the study region, site selection and field study have all been described in previous studies^67,68,70^. For the stratification, we used Fourier processed satellite imagery of the maximum normalized difference vegetation index^71^ (NDVI) and land cover data from Corine^72^ (all 1 × 1 km resolution). Our study region was limited to southern Scandinavia including all of Denmark, southern Norway and south-eastern Sweden (see Figs. 1 and 2 in Kjær et al.^70^).

**Figure 2:**
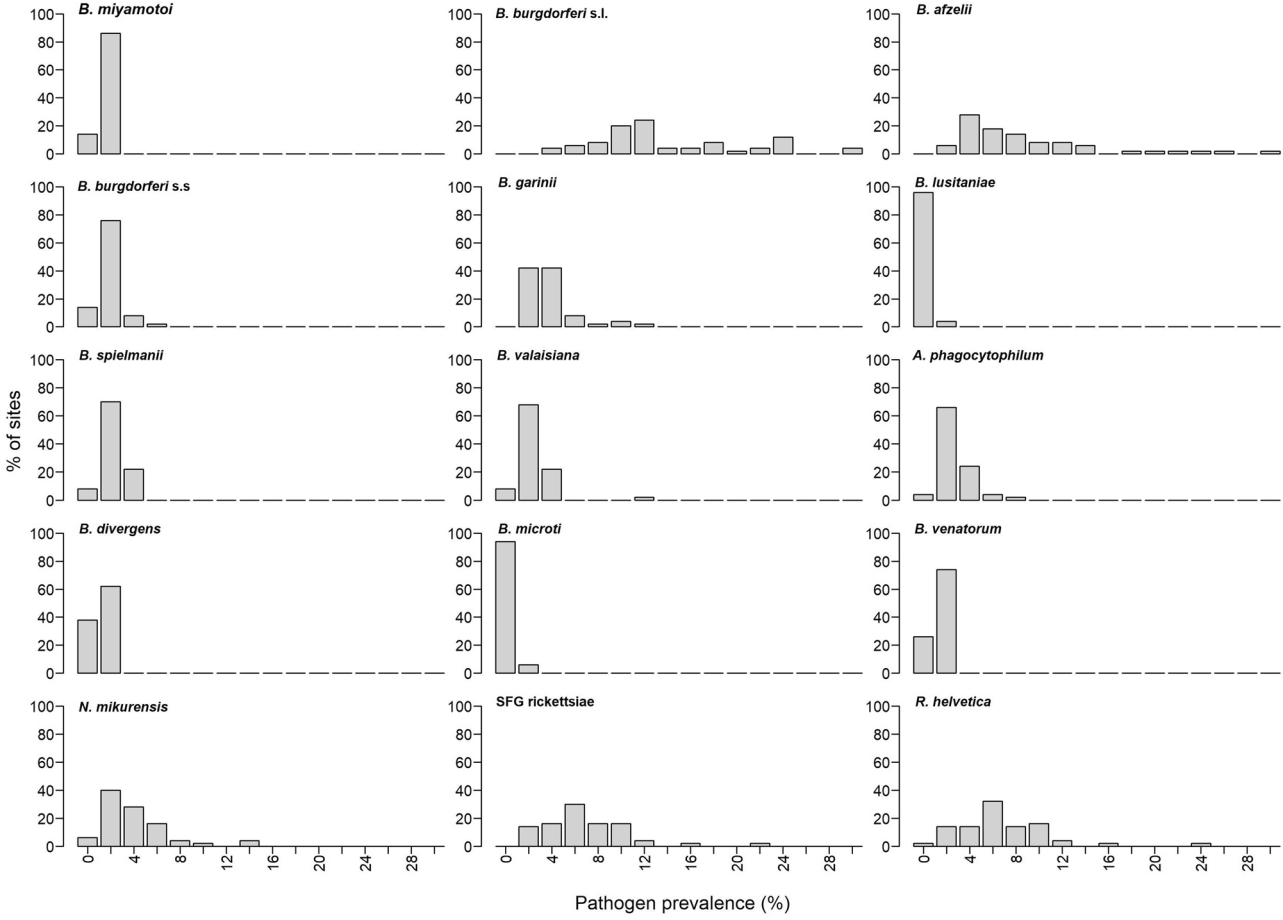
Pathogen prevalence ranges. Percentage of sites (50 total) having different ranges of individual pathogen prevalence for the 15 pathogens found within the study region.

We randomly selected 30 sites (80% forest and 20% meadow) in Denmark, 30 sites in Norway and 30 sites in Sweden for tick collection. Each of these sites had alternative sites, in case ticks could not be collected at the original site. We furthermore added 20 random sites (with 10 alternatives) along the Oslo Fjord in Norway, as this was a region of interest. We collected tick nymphs at each site between 15. August to 30. September, 2016 and stored the collected nymphs on dry ice. In the laboratory, we stored all ticks at − 80 °C until use. For a more thorough description of site selection and collection method see Kjær et al.^70^.

## Laboratory methods

### DNA extraction and screening of tick-borne pathogens by real-time PCR

Methods for DNA extraction and pathogen screening have been thoroughly described elsewhere^4,28,69^, but a short description follows. We aggregated tick nymphs into pools of 10, washed and homogenized them, and then isolated genomic DNA using the Maxwell 16 LEV Blood DNA kit (Promega, Madison, Wisconsin, USA) on a Maxwell16 Instrument. We used the BioMark real-time PCR system (Fluidigm, San Francisco, California, USA) for high-throughput microfluidic real-time PCR. We screened for bacterial and parasitic tick-borne pathogens previously found in *I. ricinus* from Scandinavia, as well as some of the most common tick-borne pathogens found in Europe: *B. miyamotoi*, *B. burgdorferi* sensu lato, *B. afzelii*, *B. burgdorferi* sensu stricto, *B. garinii*, *B. lusitaniae*, *B. spielmanii*, *B. valaisiana*, *A. phagocytophilum*, *N. mikurensis*, The spotted fever group (SFG) rickettsiae, *R. helvetica*, *Francisella tularensis*, *Coxiella burnetii*, *B. canis*, *B. divergens*, *B. microti*, *B. venatorum*, *and Bartonella henselae*^4^. The identity of the tick species *I. ricinus*, *I. persulcatus* and *Dermacentor reticulatus* was confirmed by molecular methods. We analysed data using the Fluidigm real-time PCR analysis software to obtain crossing point (CP) values, and set cut-off values to CP ≤ 28. On each chip, we included one negative water control as well as *Escherichia coli* primers and probes for internal inhibition control^4^. The Fluidigm real-time PCR method has been validated through several studies^4,22,28,73–75^.

### Statistical analysis

#### Pathogen prevalence

We calculated pool prevalence as the number of positive pools (10 nymphs per pool) out of the total number of pools for each pathogen at each site. To test for significant differences in pathogen pool prevalence between sites, we used Pearson’s chi-squared test statistics (test of equal or given proportions). We furthermore ran a mixed model logistic regression on the 2,944 pools with site as a random effect, to test for differences between countries and between strata. We used R 3.5.2^76^ for all statistical analyses.

The minimum infection rate (MIR) is often reported in pooled prevalence studies and is the number of positive pools divided by the total number of ticks tested. MIR is however, not only dependent on the true pathogen prevalence but also on the arbitrarily chosen pool size. With increasing pools sizes and pathogen prevalence, MIR will increasingly underestimate the true individual-level prevalence as positive pools will be increasingly likely to include more than one positive tick. Nevertheless, as pool size was constant for all sites (10 nymphs) in this study, MIR may simply be calculated by dividing pool prevalence with pool size.

#### Individual pathogen prevalence

We estimated the individual-level pathogen prevalence in tick nymphs at each site based on the number of positive pools and number of examined pools using method 3 from Cowing et al.^77^ that assumes 100% test sensitivity and specificity and fixed pool size. Exact confidence limits (CIs) were calculated based on binomial theory^77^.

#### Spatial analyses

To identify geographical clustering of pathogen prevalence we ran a global spatial autocorrelation test for each pathogen in ArcMap 10.6.1^78^ (Global Moran’s I). In our case, Global Moran’s I measures spatial autocorrelation based on site location and the prevalence at the sites, and evaluates whether the global prevalence patterns observed are significantly clustered, dispersed or random. Prior to analysis the geographical coordinates of each site were transformed into a flat UTM projection (all coordinates were forced into UTM zone 32 N).

Furthermore, we used the program SatScan^79^ and the rsatscan^80^ package in R 3.5.2^76^ to identify potential local pathogen clusters. For each of the pathogens, we performed spatial scan statistics with a circular to elliptic scanning window, using the Bernoulli probability model^81^ and a maximum spatial window size of less than or equal to 50% of the total population at risk. The analysis will look for significant geographical clusters where sites included in circular or ellipsoid areas on average have either higher (hotspots) or lower (coldspots) prevalence compared to the sites outside the ellipsoids. SatScan then calculates the relative risk as the estimated risk within the cluster divided by the estimated risk outside the cluster. To evaluate the clusters, we used the Gini coefficient^82^, which is a measure of heterogeneity of the collection of clusters. The Gini coefficient aids in deciding which clusters to report, by determining whether there is evidence for multiple smaller clusters or a large joint cluster. For this analysis we looked at the observed number of positive pools out of the total number of pools tested and not the estimated prevalence in individual nymphs. Again, we transformed our coordinates into a flat UTM projection (UTM zone 32 N) before running SatScan.

#### Spatial modelling

We used R 3.5.2^76^ (package caret^83^) to run different ML algorithm models on our site-specific pathogen prevalence data. We ran boosted regression tree (BRT) models (both Gaussian and Poisson loss functions) as well as support vector regression (SVR), using variables from among 92 environmental predictors as well as additional predictor variables depicting fragmentation and amount of habitat edge for our forest and meadow sites. A thorough description of the predictor variables are found in Kjær et al.^68^.

BRT uses regression trees and gradient boosting^84^, whereas SVR is based on the support vector machine method that creates hyperplanes to separate data into classes. SVR tries to fit the error rate within a certain threshold from the hyper plane^85^. For the Gaussian BRT and the SVR, we transformed our pool prevalence data using the arcsine square root transformation. For the Poisson BRT we used the number of positive pools as the dependent variable, but added the total amount of pools as a weight. We ran a Pearson correlation test to remove predictor variables correlated more than 75%. As SVRs are more sensitive than BRTs regarding the amount of variables compared to the amount of data points, we furthermore ran SVRs with recursive feature elimination^86^, leaving us with the most important model predictors for each pathogen.

For both the BRT and SVR models, we ran leave-one-out cross-validation (LOOCV) to validate our models. LOOCV is a method, where a model is fit while withholding one data point at a time, and the withheld “unknown” data points are then predicted by the model and thus used to evaluate model performance. LOOCV is often used when the sample size is too small to create a separate test set or to perform k-fold cross validation^87^. The LOOCV gives us an estimate of the prediction error in the form of the root-mean-square error, which we then normalized by dividing with the standard deviation of the observed data, resulting in a normalized root-mean-square error (NRMSE). For both the BRT and SVR models, we used a tuning grid to optimize model parameters, but held the number of trees for the BRT models constant at 1500 trees. If any of the final models proved to have suitable predictive power, they were used to predict and map prevalence to the entire study region. To draw the predictive maps, we used ArcMap 10.6.1^78^.

## Results

### Field study

Due to logistic reasons and time restraints, we collected 29,440 tick nymphs from a total of 50 sites with 30 sites in Denmark, 11 sites in Norway and 9 sites in Sweden (Fig. 2 in Kjær et al.^70^). Coordinates of these sites and number of ticks collected are available from the figshare repository^69^, and the dataset has been described in Kjær et al.^70^.

### Pathogen prevalence

In this study, a PCR run was deemed valid when all *E. coli* controls were positive, all water controls were negative, all amplification curves were accepted by the Fluidigm software algorithm for ideal curves, and CP values were less than or equal to 28. All 2,944 pools tested positive for *I. ricinus* only^17^. *F. tularensis*, *C. burnetii*, *B. canis*, or *B. henselae* were not detected in any of the 2,944 pools.

Data on presence/absence of pathogens for each pool within a site are available from the figshare repository^69^. Each of the 50 sites tested positive for at least one of the 15 pathogens identified in the survey (including *B. burgdorferi* s.l. and SFG rickettsiae) at varying prevalence. Each site had an overall pool prevalence of at least 33% (pools positive for one or more pathogens). The pathogens found were (in order of highest to lowest overall mean prevalence) *B. burgdorferi* s.l. (mean pool prevalence = 69.9%, range: 30–96.7%), *B. afzelii* (mean pool prevalence = 48.2%, range: 3.3–96.7%), *R. helvetica* (mean pool prevalence = 41.1%, range: 0.0–91.7%), SFG rickettsiae (mean pool prevalence = 41.6%, range: 1.7–90.0%), *B. garinii* (mean pool prevalence = 23.5%, range: 1.7–68.3), *N. mikurensis* (mean pool prevalence = 23.3%, range: 0.0–75.0%), *A. phagocytophilum* (mean pool prevalence = 14.1%, range: 0.0–50.0%), *B. valaisiana* (mean pool prevalence = 12.2%, range: 0.0–70.0%), *B. spielmanii* (mean pool prevalence = 11.3%, range: 0.0–30.0%), *B. burgdorferi* s.s. (mean pool prevalence = 8.5%, range: 0.0–40.0%), *B. miyamotoi* (mean pool prevalence = 4.0%, range: 0.0–16.7%), *B. venatorum* (mean pool prevalence = 3.2%, range: 0.0–10.0%), *B. divergens* (mean pool prevalence = 1.8%, range: 0.0–6.9%)*, B. microti* (mean pool prevalence = 0.1%, range: 0.0–1.7%), and *B. lusitaniae* (mean pool prevalence = 0.1%, range: 0.0–3.3%).

The Pearson’s chi square test, to test for equality of proportions among the sites, showed significant differences in prevalence between sites for all pathogens except the three relatively rare *Babesia* species—*B. divergen*s, *B. venatorum*, and *B. microti* (Table 1).

**Table 1:** Pearson's chi square test for equality of proportions without continuity correction, testing for significant differences in pathogen prevalence (pool prevalence) between the 50 sites in Denmark, Norway and Sweden.

Pathogen	χ^2^	Df	*P* value
*B. miyamotoi*	83.27	49	< 0.01
*B. afzelii*	655.92	49	< 0.0001
*B. burgdorferi* s.s	241.57	49	< 0.0001
*B. garinii*	399.29	49	< 0.0001
*B. lusitaniae*	78.86	49	< 0.01
*B. spielmanii*	209.84	49	< 0.0001
*B. valaisiana*	364.36	49	< 0.0001
*A. phagocytophilum*	348.04	49	< 0.0001
*B. divergens*	61.04	49	0.12
*B. microti*	46.67	49	0.57
*B. venatorum*	65.69	49	0.056
*N. mikurensis*	555.36	49	< 0.0001
*R. helvetica*	483.17	49	< 0.0001

Only tick-borne pathogens identified from *I. ricinus* ticks collected from Denmark, Norway and Sweden, 2016 are depicted (excluding *B. burgdorferi* s.l. and SFG rickettsiae).

The mixed model logistic regression showed that only pool prevalence of *B. miyamotoi* had an effect of stratum when taking site variation into account. Here, prevalence differed significantly between all strata (*P* < 0.001) with higher values in strata with low NDVI. Meadow sites with low NDVI had the highest pool prevalence. We also tested for differences between countries and found significantly higher prevalence in Norway compared to Denmark and Sweden for *B. burgdorferi* s.l., *B. afzelii*, and *N. mikurensis* (all *P* < 0.001). This pattern was reversed for *R. helvetica* and SFG rickettsiae, with prevalence being significantly lower in Norway than in Denmark and Sweden (*P* < 0.001).

### Individual prevalence

An overview of the estimated specific pathogen prevalence in individual nymphs and frequencies are found in Figs. 1 and 2. All estimated site-level individual prevalence and CI’s are available from the figshare repository^69^. Table 2 describes the overall mean and range of all the pathogens found in this study. Overall, the *B. burgdorferi* sensu lato complex had the highest mean prevalence (13.0%), and was found at all sites. *B. afzelii* contributed mostly to this pattern with a mean prevalence of 7.9% (all sites testing positive), followed by *B. garinii* with 2.9% (all sites testing positive), *B. valaisiana* with 1.4% (positive at 92% of the sites), *B. spielmanii* with 1.2% (positive at 92% of the sites), and *B. burgdorferi* s.s. with a mean prevalence of 0.9% (positive at 86% of the sites). *B. lusitaniae* was only found at two sites, both in Denmark, in both cases with low prevalence (0.2% and 0.3%). *B. miyamotoi* had a mean prevalence of 0.4% *(*positive at 86% of the sites), and mean prevalence for *A. phagocytophilum* was 1.6% (positive at 96% of the sites). Mean prevalence was 0.2% for *B. divergens* (positive at 62% of the sites), and 0.3% for *B. venatorum* (positive at 74% of the sites). *B. microti* was found with low prevalence at three sites in the Skåne region in Sweden (all 0.2%). *N. mikurensis* had a mean prevalence of 3.0% (positive at 94% of the sites), with 5.8% for SFG rickettsiae (positive at 100% of the sites), and 5.8% for *R. helvetica* (positive at 98% of the sites).

**Table 2:** Average and range of pathogen prevalence (estimated individual nymph prevalence) in southern Scandinavia.

Pathogen	Average (%)	Range (%)
*B miyamotoi*	0.4	0.0–1.8
*B. burgdorferi* s.l	13.0	3.5–28.8
*B. afzelii*	7.9	0.3–28.8
*B. burgdorferi* s.s	0.9	0.0–5.0
*B. garinii*	2.9	0.2–9.1
*B. lusitaniae*	0.01	0.0–0.3
*B. spielmanii*	1.2	0.0–3.5
*B. valaisiana*	1.4	0.0–11.3
*A. phagocytophilum*	1.6	0.0–6.7
*B. divergens*	0.2	0.0–0.7
*B. microti*	0.01	0.0–0.2
*B. venatorum*	0.3	0.0–1.1
*N. mikurensis*	3.0	0.0–12.9
SFG rickettsiae	5.8	0.2–20.6
*R. helvetica*	5.8	0.0–22.0

Only the 15 tick-borne pathogens identified from *I. ricinus* ticks collected from Denmark, Norway and Sweden, 2016 are depicted (including *B. burgdorferi* s.l. and SFG rickettsiae).

### Spatial analysis

Results from the global Moran’s I only identified *B. afzelii*, *B. microti*, *N. mikurensis* and *R. helvetica* as having a clustered prevalence patterns where neighbouring sites tended to have more similar prevalence than expected from a random distribution (*P* < 0.05, Table 3). The global prevalence patterns of the remaining pathogens did not appear to differ significantly from a random geographical pattern (Table 3).

**Table 3:** Global Moran’s I test, testing for spatial autocorrelation of pathogen prevalence (pool prevalence) between the 50 sites in Denmark, Norway and Sweden.

Pathogen	Moran’s index	z-score	*P* value
*B. miyamotoi*	− 0.01	0.08	0.93
*B. afzelii*	0.34	4.85	< 0.0001
*B. burgdorferi* s.s	0.10	0.62	0.10
*B. garinii*	− 0.03	− 0.07	0.94
*B. lusitaniae*	− 0.02	0.03	0.98
*B. spielmanii*	− 0.08	− 0.79	0.43
*B. valaisiana*	− 0.08	49	0.33
*A. phagocytophilum*	0.01	0.43	0.67
*B. divergens*	− 0.07	− 0.63	0.53
*B. microti*	0.14	2.45	0.014
*B. venatorum*	− 0.03	− 0.14	0.89
*N. mikurensis*	0.36	5.11	< 0.0001
*R. helvetica*	0.47	6.68	< 0.0001

Only tick-borne pathogens identified from *I. ricinus* ticks collected from Denmark, Norway and Sweden, 2016 are depicted (excluding *B. burgdorferi* s.l. and SFG rickettsiae).

The SatScan local spatial analysis detected significant clusters within the region for the *B. burgdorferi* s.l. complex, hereunder for *B. afzelii, B. burgdorferi* s.s., *B. garnii, B. spielmanii,* and *B. valaisiana* (Fig. 1, Supplementary Table S1 online). The analysis also detected significant clusters for *A. phagocytophilum, N. mikurensis*, and SFG rickettsiae and *R. helvetica*. Prevalence hot spots and cold spots were interspersed for most of the pathogens, with no obvious interpretable overall geographical patterns. For *B. burgdorferi* s.s., *B. spielmanii*, *N. mikurensis* and *R. helvetica* some clusters even connected sites, separated by large water bodies, further indicating that no clear patterns were discernible. The number of sites within hot spots ranged from 1–11, and ranged from 1–15 within cold spots (Supplementary Table S2 online).

### Spatial modelling

We created BRT and SVR models for 13 of the 15 pathogens found in the region (including *B. burgdorferi* s.l. and SFG rickettsiae). *B. lusitaniae* and *B. microti* were both highly zero-inflated as only two and three sites were positive for these pathogens respectively, and were thus omitted from the analyses. Removing highly correlated variables (> 75%) resulted in 56 predictors used in both the BRT and SVR models. NRMSE, and R^2^-values for all final best models can be found in Table 4. Except for *R. helvetica* and *N. mikurensis*, the pathogen models generally performed poorly with NRMSE-values > 0.85 and R^2^-values ranging from 0.002–0.24, and were consequently not used to create prediction maps for the region. In general, the SVR models performed better than the BRT models and the BRT models with Gaussian loss performed better that the models with Poisson loss. Tuning grid values for all final best models can be found in the Supplementary Table S2 online.

**Table 4:** Comparison of the best SVR and BRT models (lowest NRMSE) for each of the 13 pathogens (excluding *B. microti* and *B. lusitaniae*).

Pathogen	SVM model type	R^2^	NRMSE	# variables	BRT model	R^2^	NRMSE
*B. miyamotoi*	Polynomial kernel	0.07	1.01	65^a^	Poisson	0.06	1.17
*B. burgdorferi* s.l	Linear kernel	0.22	0.89	65	Gaussian	0.05	1.05
*B. afzelii*	Polynomial kernel	0.24	0.89	40	Gaussian	0.23	0.89
*B. burgdorferi* s.s	Linear kernel	0.07	0.96	65^a^	Gaussian	0.11	0.96
*B. garinii*	Polynomial kernel	0.03	0.99	50	Gaussian	0.05	1.25
*B. spielmanii*	Radial kernel	0.00	0.97	65^a^	Gaussian	0.15	1.29
*B. valaisiana*	Radial kernel	0.21	1.03	65^a^	Gaussian	0.01	1.10
*A. phagocythophilum*	Radial kernel	0.19	0.90	5	Gaussian	0.04	1.04
*B. divergens*	Polynomial kernel	0.08	0.95	20	Gaussian	0.02	1.19
*B. venatorum*	Polynomial kernel	0.17	0.93	30	Gaussian	0.17	0.92
*N. mikurensis*	Polynomial kernel	0.43	0.75	10	Gaussian	0.29	0.84
SFG rickettsiae	Linear kernel	0.45	0.72	5	Gaussian	0.40	0.77
*R. helvetia*	Linear kernel	0.48	0.74	10	Gaussian	0.41	0.76

NRMSE is the normalized root-mean-square error (root-mean-square error divided by the standard deviation).

^a^Both SVR and BRT methods transform factorial predictors into dummy variables, resulting in more predictors than the 56 predictors originally entered in the models.

**Figure 3:**
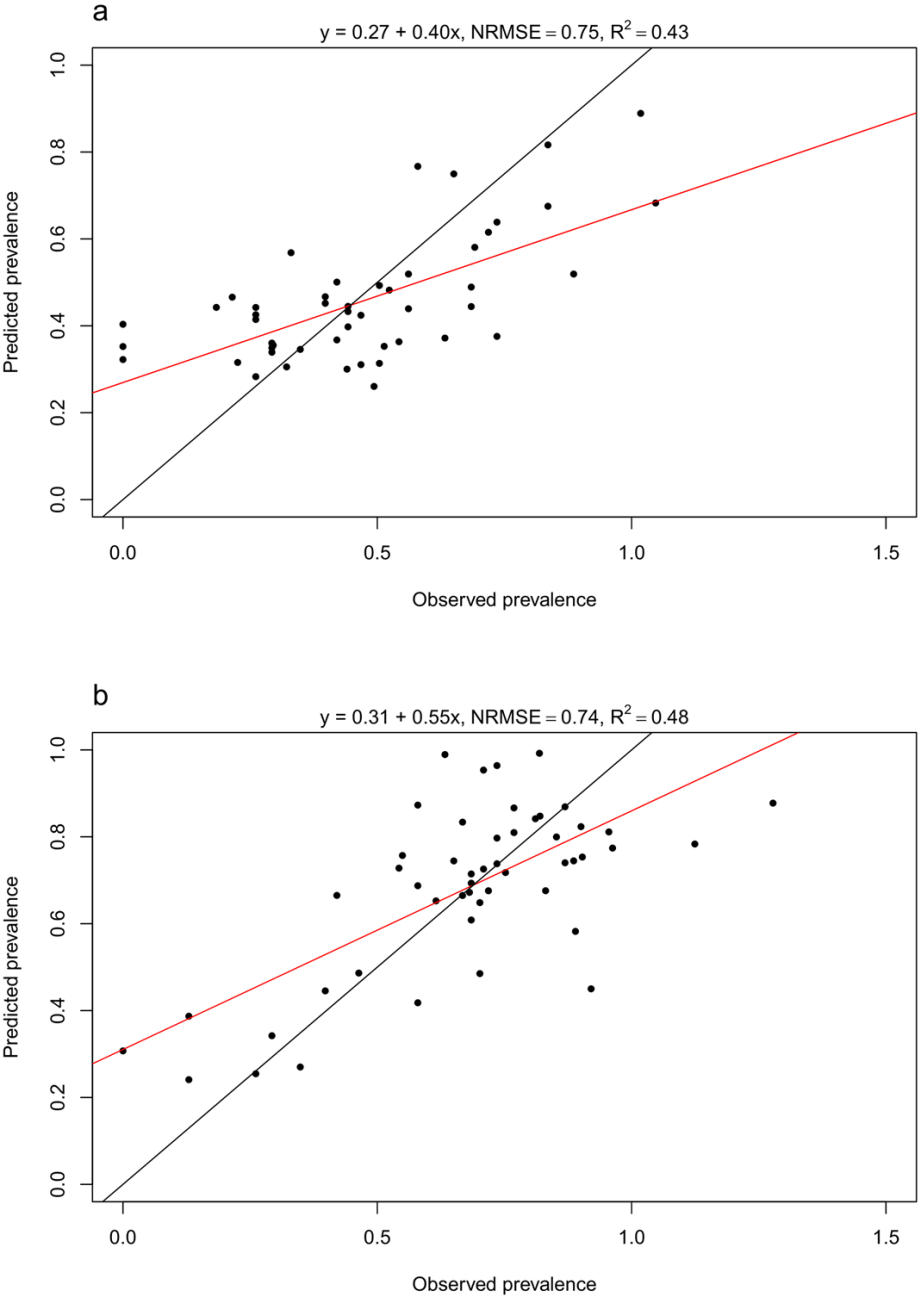
Observed versus predicted prevalence. Observed prevalence plotted against predicted prevalence (pool prevalence, both arc-sine-square-root transformed) for (**a**) *N. mikurensis* and (**b**) *R. helvetica*, based on LOOCV results from the final best SVR models. The red line is a linear regression line on the observed and predicted values, and the black line depicts a 1:1 relationship between observed and predicted values. NRMSE is the normalized root-mean-square error.

**Figure 4:**
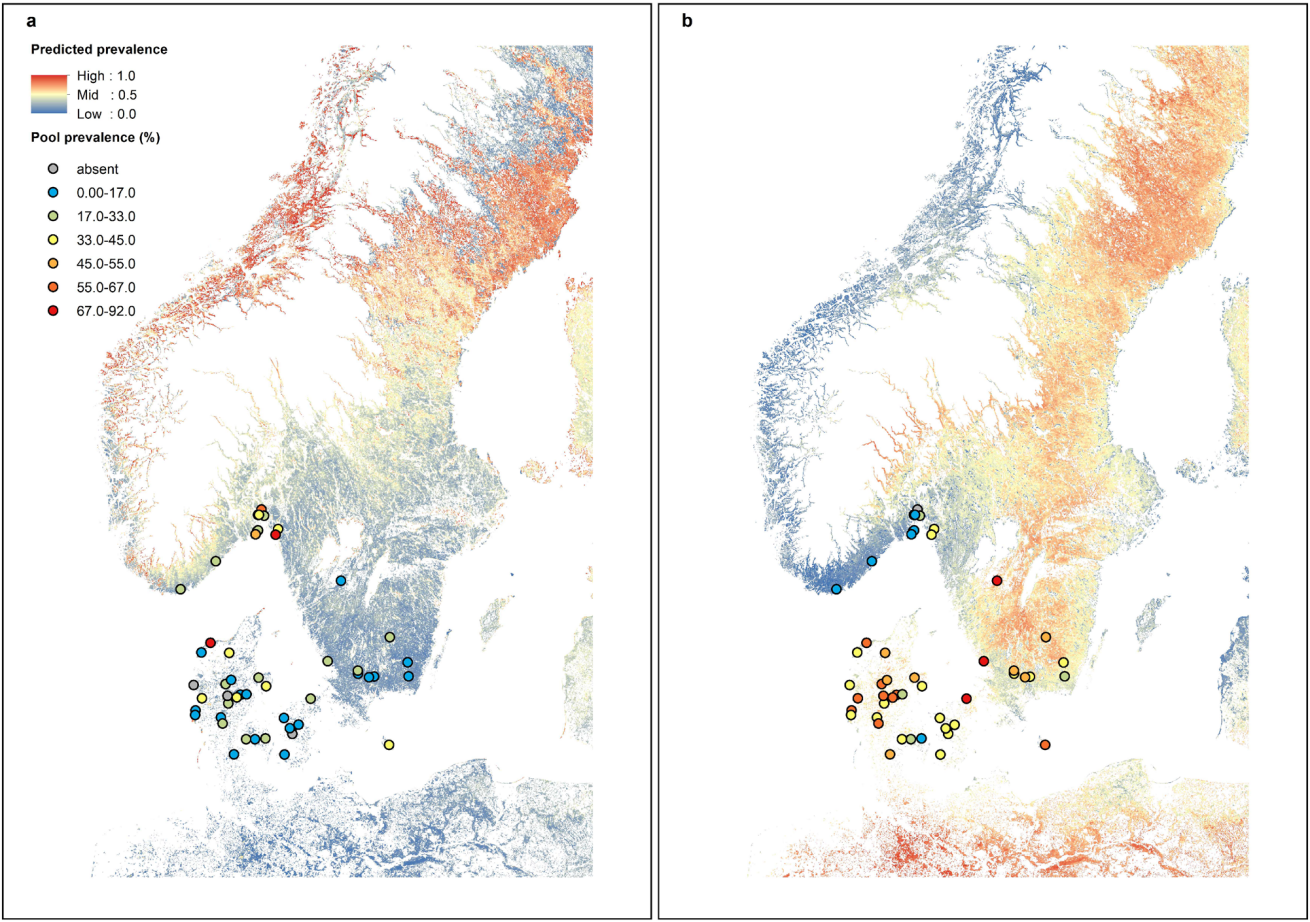
Prevalence maps. Maps of predicted prevalence (pool prevalence, back-transformed from arc-sine-square-root) for (**a**) *N. mikurensis* and (**b**) *R. helvetica* from the final SVR models. Observed pool prevalence at the 50 study sites is also depicted. White areas are altitudes above 450 m or lakes, rivers and streams, or habitats other than forest or meadow (not predicted). The maps were created using ArcMap 10.6.1^78^.

The best and final models for *N. mikurensis* and *R. helvetica* were the SVR models with polynomial and linear kernels respectively. Both of these models ended up with a total of ten predictors, selected via the recursive feature elimination method (See Supplementary Figs. S1 and S2 online). The models resulted in NRMSE values of 0.75 and 0.74 and R^2^ values of 0.43 and 0.48, respectively (Fig. 3). We plotted the prediction errors for both models (observed pool prevalence – predicted pool prevalence, both arcsine-square-root transformed) for the LOOCV predictions, to check for potential spatial patterns, and did not observe any clear patterns (See Supplementary Fig. S3 online). The final prediction maps included all of Denmark, 68.4% of Norway and 85.8% of Sweden (Fig. 4). The maps show relatively high prevalence of *N. mikurensis* in the north-eastern parts of Sweden as well as the eastern and north-eastern coastline of Norway. *R. helvetica* prevalence was high throughout Denmark and Sweden, but low in Norway, except the northern parts of the Oslo Fjord.

The ten predictors driving both models were daytime temperature and related parameters, the vegetation indexes NDVI and EVI and their related parameters, and parameters related to the middle infra-red index (See Supplementary Figs. S1 and S2 online). In the model for *N. mikurensis*, land cover ranked in 4th place, but mostly due to moors and heathland (See Supplementary Fig. S1 online).

## Discussion

We tested 29,440 *I. ricinus* nymphs for 19 different pathogens in 2,944 pools (including *B. burgdorferi* s.l. and SFG rickettsiae). We found a high pathogen infection in the collected ticks, corresponding to what has been found for the region in other studies^4,15,23,25,28,54,88–90^ . Previous studies have found overall prevalence of the *B. burgdorferi* s.l. complex ranging from 10–15% in tick nymphs from recorded studies in Denmark, Norway and Sweden^15,26,89–92^, with local prevalence as high as 33.1% in southern Norway^27^. In our study, estimated individual prevalence over 20% were found along the Oslo Fjord in Norway, and at two sites in Jutland and one on Zealand in Denmark. *B. lusitaniae* was only found at two sites in Denmark, Jutland and Bornholm, but has previously been reported from northern Zealand in Denmark^26^, and Östergötland in Sweden^90^. Our estimated prevalence for *A. phagocytophilu*m was also within the recorded prevalence range of 1–24% in tick nymphs for the region^15,23,33,89,93^. The overall prevalence for the *Babesia* spp. were ≤ 1% whereas other studies from the region have reported prevalence of *Babesia* spp. up to 3.6%^4,28,94,95^. Reported prevalence in tick nymphs for *N. mikurensis* have ranged from 0–19% for the region^4,15,28,31,50,96,97^, with high prevalence in southern Norway, particularly along the Oslo Fjord^15^. Our estimated prevalence was well within the reported range, also with high local prevalence along the Oslo Fjord in Norway. All estimated prevalence for *N. mikurensis* higher than 5% was found along the Oslo Fjord and in Jutland in Denmark. A study from 2015 recorded *R. helvetica* for the first time in Norway in one sample of adult ticks^89^, and has since been found to be widespread at a low prevalence in southern Norway (Kjelland, unpublished data). *R. helvetica* has previously been recorded in tick nymphs from Denmark and Sweden with prevalence ranging from to 1.4–18%^93,98^. Our prevalence was within that range except for a prevalence of 22% at one site in Sweden. Interestingly, we found *R. helvetica* at all sites except one site in Norway, supporting that although *R. helvetica* was detected only recently in Norway, the pathogen is common in southern Norway at prevalence ≤ 5%. The significantly lower pool prevalence of *R. helvetica* in Norway compared to Denmark and Sweden, and the SatScan analysis showing a cold spot for *R. helvetica* in Norway, could also suggest that the presence of *R. helvetica* in Norway may be of newer origin. We did not find *F. tularensis*, *B. canis* or *B. henselae* at any of our sites, however our survey does not rule out the presence of these pathogens in the study area albeit at low prevalence^17^. *B. canis* is transmitted via the meadow tick (*D. reticulatus*)^99^, and as all our collected ticks were *I. ricinus*, we did not expect to find this pathogen.

Except for the three *Babesia* species, we found a significant difference in pathogen prevalence between sites. These differences indicate that environmental factors may be driving the different prevalence at the specific sites. We sampled all sites within the same period using the same field protocol, thus any difference in prevalence should be a result of some form of causality. The pathogens with no differences between sites generally also had very low prevalence and thus low statistical power to detect potential differences between sites. This especially applies to *B. microti* which is considered absent from most of the study area except southern Sweden^94^ where we also found it in this study. The interplay between ticks, host species and the environment is complex, but finding drivers of pathogen prevalence may aid in creating future predictive models and risk maps for unsampled regions. However, it can be challenging to determine drivers of the different pathogens, causing the observed patterns. Some of our sites may have differed in the abundance of rodent species, causing differences in the prevalence of several pathogen species having rodents as reservoirs^45,52^. Sites with relatively high abundance of different bird species could explain prevalence patterns of *B. garinii* and *B. valaisiana*^19,45^, and the presence and abundance of cervids at our sites could impact the prevalence of *B. venatorum*^45^. *R. helvetica, A. phagocytophilum* and possibly *N. mikurensis* may have a broader spectrum of host species^18,31,47,53^, which could explain why we found these pathogens throughout the region.

To further look for causal effects, we conducted two geographical cluster analyses. The Global Moran’s I showed significant clustering for *B. afzelii*, *B. microti* (only two sites were positive for this pathogen), *N. mikurensis* and *R. helvetica*, suggesting that nearby sites had similar suitability for these pathogens. *B. microti* has recently been reported from the central part of southern Sweden but was never reported in Denmark and Norway, suggesting this area may be a hot spot for *B. microti* in Scandinavia. We furthermore investigated whether we could identify clusters of neighbouring sites where prevalence was significantly higher or lower compared to surrounding areas. We were expecting some form of overall latitudinal or environmental gradient that correlated with the distribution of key environmental or climatic variables. The geographical clusters we found however, did not show a clear interpretable pattern, with hotspots and cold spots of prevalence dispersed throughout the region. Previous studies have found significant effects of environmental variables (temperature, NDVI, land cover) on the spatial variation in the occurrence of TBEV^65,100,101^. Transmission of TBEV is thought to be dependent on co-feeding of larvae and nymphs on hosts, and thus the synchrony of the two instars^100,102,103^. This synchrony is determined by the tick life cycle which in turn is affected by environmental conditions^12,100^. The transmission of bacteria and parasites, however, may be more dependent on the availability of competent host species and factors affecting this availability^47,100,104^. While our two spatial cluster analyses proved that there is significant geographical clustering for some of the pathogens analysed, we were not able to discern any obvious single predictors associated geographically with these clusters.

The lack of discernible predictors could explain why our predictive ML pathogen models had poor predictive power. As tick abundance is thought to affect pathogen prevalence^54,55,57^, we tried to run our ML models with observed abundance of different tick instars at each site^68^ (data not presented), however, this did not improve the models, and these abundance predictors were thus left out of the final models. Only the models for *N. mikurensis* and *R. helvetica* had moderate predictive power and were the only models used to create predictive maps. Abundance and/or diversity of host species for these two pathogens may be affected by environmental variables that could act as proxies in the models. The models for both *N. mikurensis* and *R. helvetica* were driven by temperature and vegetation parameters and when comparing predicted prevalence to the actual prevalence data (Fig. 4), the models predicted well for known areas in Denmark and the southern parts of Norway and Sweden. We did not test ticks from northern Sweden and north-eastern Norway, thus predictions for these areas should be interpreted with caution. However, Jenkins et al.^50^ found *N. mikurensis* prevalence of ca. 6.5% in the north-western parts of Norway, thus our models may still be useful for these areas. Both prediction maps show high predicted prevalence of both *N. mikurensis* and *R. helvetica* in northern Sweden above the biogeographical and climatic boundary called Limes Norrlandicus (LN)^105^. In our previous predictive modelling studies^67,68^, we found that LN reflected boundaries for tick distribution and abundance, and thus predictions of high pathogen prevalence in areas above this boundary are questionable. Low geographical spread of our data in general could explain the low predictive power of most of our pathogen models. Low sample size could also explain the low model performances, as a low number of data points with potential low variability in the predictors may not be sufficient for the ML methods to learn patterns and dependencies between the data and the predictors. The use of LOOCV may additionally lead to overfitting and thus low generalisability to unseen data, reducing the predictive power. Poor model performance could also be due to absence of key pathogen drivers such as data on host species composition and abundance, which our environmental data could not act as proxies for. As data on host species can be hard to obtain at a high resolution, it may complicate predictive modelling of certain pathogens. We do not know how stable the observed prevalence is and if the pattern we found in 2016, would also be observed in later years. If the differences we observed in 2016 are not due to differences in host species composition (which can also fluctuate between years) or other potential drivers, but instead are due to temporal fluctuations and epidemics in the wild hosts, it will complicate predictive modelling and mapping.

## Conclusion

We found high prevalence of tick-borne pathogens in tick nymphs from southern Scandinavia. Particularly *Borrelia* spp., *N. mikurensis*, and *R. helvetica* were widespread throughout the region. Although *R. helvetica* has only recently been found in Norway, we found that the pathogen was common throughout Norway, albeit at prevalence ≤ 5%. Significant differences in prevalence between sites and geographically interspersed clusters with high and low prevalence, suggest highly complex patterns, which complicates creating predictive models of pathogen prevalence.

## Supplementary information

Supplementary Information.

## Data Availability

All data generated or analysed during this study are available on figshare (https://doi.org/10.6084/m9.figshare.c.4938270.v1).
