# Supplementary material for: Spatial patterns of pathogen prevalence in questing Ixodes ricinus nymphs in southern Scandinavia, 2016
Source: Sci Rep. 2020 Nov 9;10:19376. doi: 10.1038/s41598-020-76334-5 (PMC7652892; doi:10.1038/s41598-020-76334-5)
Supplement: Supplementary file 1 — Supplementary Information. [file 41598_2020_76334_MOESM1_ESM.docx]

# Spatial patterns of pathogen prevalence in questing *Ixodes ricinus* nymphs in southern Scandinavia, 2016

**(Scientific Reports, Supplementary information)**

Lene Jung Kjær^1*^, Kirstine Klitgaard^2^, Arnulf Soleng^3^, Kristin Skarsfjord Edgar^3^, Heidi Elisabeth H. Lindstedt^3^, Katrine M. Paulsen^4,5^, Åshild Kristine Andreassen^4^, Lars Korslund^6^, Vivian Kjelland^6,7^, Audun Slettan^6^, Snorre Stuen^8^, Petter Kjellander^9^, Madeleine Christensson^9^, Malin Teräväinen^9^, Andreas Baum^10^, Laura Mark Jensen^1^, and René Bødker^1^

^1^ Department of Veterinary and Animal Sciences, Faculty of Health and Medical Sciences, University of Copenhagen, Frederiksberg, Denmark

^2^Department for Diagnostics and Scientific Advice, National Veterinary Institute, Technical University of Denmark, Lyngby, Denmark

^3^Department of Pest Control, Norwegian Institute of Public Health, Oslo, Norway

^4^Department of Virology, Norwegian Institute of Public Health, Oslo, Norway

^5^Department of Production Animal Clinical Sciences, Norwegian University of Life Sciences, Oslo Norway

^6^Department of Natural Sciences, University of Agder, Kristiansand, Norway

^7^Sørlandet Hospital Health Enterprise, Research Unit, Kristiansand, Norway

^8^Department of Production Animal Clinical Sciences, Section of Small Ruminant Research, Norwegian University of Life Sciences, Sandnes, Norway

^9^Department of Ecology, Grimsö Wildlife Research Station, Swedish University of Agricultural Sciences, Riddarhyttan, Sweden

^10^Department of Applied Mathematics and Computer Science, Technical University of Denmark, Lyngby, Denmark

*Corresponding author: lenju@sund.ku.dk

**Supplementary Table S1.** Results from elliptical cluster analysis performed using SatScan^1^ on the pool prevalences calculated for the tick nymphs collected from 50 sites in Denmark, Norway and Sweden, 2016. Only pathogens with significant clusters and clusters with the maximum Gini coefficient are depicted. Relative risk is calculated by SatScan as the estimated risk within the cluster divided by the estimated risk outside the cluster. LL = Log-likelihood.

| Pathogen | # sites in cluster | LL ratio | P-value | Relative risk |
| --- | --- | --- | --- | --- |
| *B. burgdorferi* s.l. | 4 | 44.7 | <0.0001 | 1.4 |
| *B. burgdorferi* s.l. | 2 | 28.5 | <0.0001 | 0.5 |
| *B. burgdorferi* s.l. | 1 | 20.9 | <0.0001 | 0.4 |
| *B. burgdorferi* s.l. | 3 | 17.0 | <0.0001 | 0.7 |
| *B. burgdorferi* s.l. | 3 | 15.1 | <0.001 | 1.3 |
| *B. burgdorferi* s.l. | 4 | 12.9 | 0.002 | 1.2 |
| *B. burgdorferi* s.l. | 5 | 11.1 | <0.01 | 0.8 |
| *B. burgdorferi* s.l. | 2 | 10.6 | 0.014 | 1.3 |
| *B. afzelii* | 3 | 74.9 | <0.0001 | 2.0 |
| *B. afzelii* | 3 | 54.0 | <0.0001 | 1.8 |
| *B. afzelii* | 2 | 52.4 | <0.0001 | 0.1 |
| *B. afzelii* | 3 | 40.9 | <0.0001 | 1.7 |
| *B. afzelii* | 3 | 24.4 | <0.0001 | 0.5 |
| *B. afzelii* | 3 | 20.0 | <0.0001 | 0.5 |
| *B. afzelii* | 4 | 19.6 | <0.0001 | 0.6 |
| *B. afzelii* | 3 | 9.6 | 0.025 | 1.4 |
| *B. burgdorferi* s.s. | 7 | 31.4 | <0.0001 | 0.0 |
| *B. burgdorferi* s.s. | 1 | 22.9 | <0.0001 | 5.1 |
| *B. burgdorferi* s.s*.* | 3 | 14.9 | <0.001 | 2.8 |
| *B. burgdorferi* s.s. | 9 | 8.7 | 0.047 | 1.8 |
| *B. garnii* | 2 | 28.7 | <0.0001 | 2.5 |
| *B. garnii* | 1 | 20.2 | <0.0001 | 2.7 |
| *B. garnii* | 1 | 20.2 | <0.0001 | 2.7 |
| *B. garnii* | 2 | 16.0 | <0.001 | 0.2 |
| *B. garnii* | 2 | 16.0 | <0.001 | 0.2 |
| *B. garnii* | 2 | 13.6 | <0.001 | 0.3 |
| *B. garnii* | 2 | 11.8 | 0.004 | 1.9 |
| *B. garnii* | 2 | 10.0 | 0.014 | 0.3 |
| *B. garnii* | 1 | 9.9 | 0.014 | 0.1 |
| *B. spielmanii* | 5 | 17.0 | <0.0001 | 0.2 |
| *B. spielmanii* | 7 | 14.9 | <0.001 | 0.3 |
| *B. spielmanii* | 2 | 14.8 | 0.001 | 0.0 |
| *B. spielmanii* | 3 | 11.5 | 0.006 | 2.2 |
| *B. spielmanii* | 6 | 11.2 | 0.008 | 1.9 |
| *B. valaisiana* | 1 | 55.7 | <0.0001 | 6.3 |
| *B. valaisiana* | 2 | 16.1 | <0.001 | 0.0 |
| *A. phagocytophilum* | 2 | 34.4 | <0.0001 | 3.5 |
| *A. phagocytophilum* | 3 | 23.4 | <0.0001 | 0.0 |
| *A. phagocytophilum* | 1 | 17.1 | <0.0001 | 3.3 |
| *A. phagocytophilum* | 3 | 14.2 | 0.001 | 0.2 |
| *A. phagocytophilum* | 2 | 13.7 | 0.001 | 2.4 |
| *A. phagocytophilum* | 3 | 10.7 | 0.011 | 0.3 |
| *A. phagocytophilum* | 3 | 9.5 | 0.028 | 0.3 |
| *N. mikurensis* | 11 | 138.3 | <0.0001 | 3.0 |
| *N. mikurensis* | 15 | 74.0 | <0.0001 | 0.3 |
| *N. mikurensis* | 2 | 32.8 | <0.0001 | 0.0 |
| SFG rickettsiae | 3 | 58.5 | <0.0001 | 2.1 |
| SFG rickettsiae | 3 | 48.3 | <0.0001 | 0.2 |
| SFG rickettsiae | 3 | 39.1 | <0.0001 | 0.3 |
| SFG rickettsiae | 3 | 20.6 | <0.0001 | 1.6 |
| SFG rickettsiae | 3 | 19.7 | <0.0001 | 0.5 |
| SFG rickettsiae | 3 | 11.3 | 0.007 | 0.6 |
| *R. helvetica* | 4 | 91.8 | <0.0001 | 0.1 |
| *R. helvetica* | 3 | 59.7 | <0.0001 | 2.1 |
| *R. helvetica* | 3 | 39.8 | <0.0001 | 0.3 |
| *R. helvetica* | 4 | 23.1 | <0.0001 | 1.6 |
| *R. helvetica* | 4 | 11.6 | 0.005 | 0.6 |

**Supplementary Table S2.** Boosted Regression Tree and Support Vector Regression tuning parameters for all the final best pathogen models, validated using leave-one-out cross validation. ID = interaction depth, LR = learning rate, MON = minimum number of observations in node.

|  | SVR parameters | | | | | BRT parameters | | |
| --- | --- | --- | --- | --- | --- | --- | --- | --- |
| Pathogen model | Kernel | C | sigma | degree | scale | ID | LR | MON |
| *B.* *miyamotoi* | Polynomial | 0.01 |  | 2 | 0.01 | 1 | 0.01 | 1 |
| *B. burgdorferi* s.l. | Linear | 0.01 |  |  |  | 3 | 0.015 | 2 |
| *B. afzelii* | Polynomial | 0.01 |  | 2 | 0.1 | 3 | 0.1 | 2 |
| *B. burgdorferi* s.s. | Linear | 0.01 |  |  |  | 3 | 0.1 | 1 |
| *B. garinii* | Polynomial | 0.1 |  | 2 | 0.01 | 2 | 0.01 | 3 |
| *B. spielmanii* | Radial | 0.1 | 0.01 |  |  | 1 | 0.01 | 5 |
| *B. valaisiana* | Radial | 0.01 | 0.01 |  |  | 3 | 0.1 | 1 |
| *A. phagocythophilum* | Radial | 1 | 0.1 |  |  | 1 | 0.01 | 5 |
| *B. divergens* | Polynomial | 0.01 |  | 3 | 0.1 | 3 | 0.02 | 1 |
| *B. venatorum* | Polynomial | 0.01 |  | 4 | 0.1 | 1 | 0.05 | 1 |
| *N. mikurensis* | Polynomial | 1 |  | 4 | 0.01 | 1 | 0.2 | 1 |
| SFG rickettsiae | Linear | 1 |  |  |  | 2 | 0.05 | 2 |
| *R. helvetia* | Linear | 1 |  |  |  | 3 | 0.1 | 5 |

**Supplementary Figure S1. Plots of the effect of the 10 predictors in the final best SVR model for *N. mikurensis*.** Only 9 predictors are shown with their partial dependence plots, which show the marginal effect of the predictors on the response after integrating out other predictors. As land cover is a factorial predictor, the SVR modelling process creates dummy variables and thus a partial dependence plot is not available. Instead, we plotted the predicted prevalence against the different land cover types in our model. All daytime temperatures are land surface temperatures.

NDVI is the Normalized difference vegetation index, EVI is the enhanced vegetation index, land cover codes are: 21 - Land principally occupied by agriculture, with significant areas of natural vegetation, 23 – Broad-leaved forest, 24 – Coniferous forest, 25 – Mixed forest, and 27 – Moors and heathland

**Supplementary Figure S2. Partial dependence plots of the 10 predictors in the final best SVR model for *R. helvetica*.** Partial dependence plots show the marginal effect of the predictors on the response after integrating out other predictors. All daytime temperatures are land surface temperatures.

NDVI is the Normalized difference vegetation index, EVI is the enhanced vegetation index.

**Supplementary Figure S3.** **Residual plots.** Observed prevalence – predicted prevalence (pool prevalence, both arc-sine-square-root transformed) of a) *N. mikurensis* and b) *R. helvetica*, based on the final SVR models to predict pathogen prevalence in southern Scandinavia. Negative values show sites with higher predicted prevalence than is observed and positive values show sites where predicted prevalence is lower than the observed prevalence. The maps were created using ArcMap 10.6.1^2^.


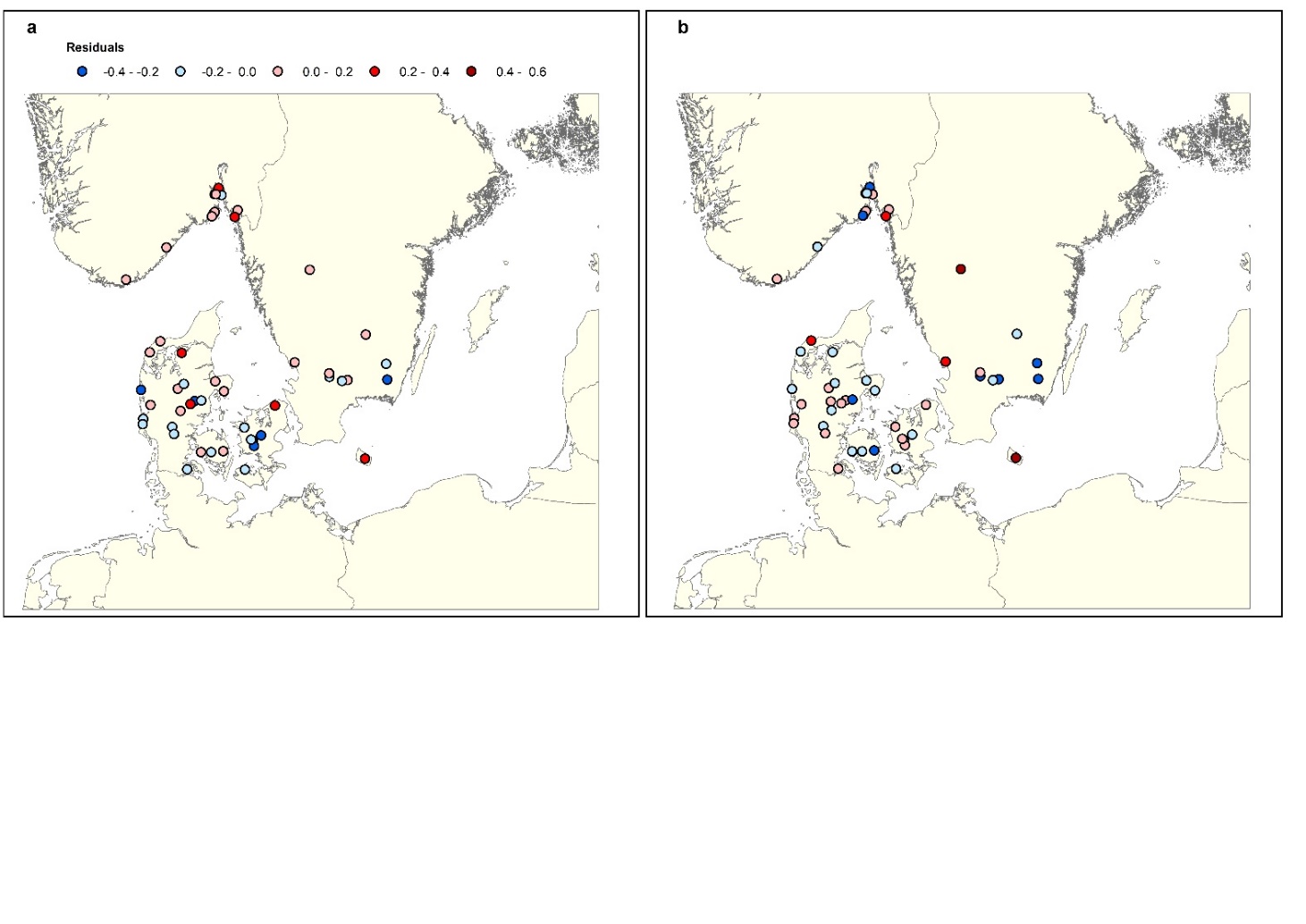


**References**

1. Kulldorff M. and Information Management Services, I. SaTScan^TM^ v9.6: Software for the spatial and space-time scan statistics. [www.satscan.org], 2018.

2. Environmental Systems Research Institute. ArcGIS Desktop: Release 10.6.1. (2017).
